# Metastasis to the gluteus maximus muscle from renal cell carcinoma with special emphasis on MRI features

**DOI:** 10.1186/1477-7819-5-88

**Published:** 2007-08-04

**Authors:** Akio Sakamoto, Tatsuya Yoshida, Suguru Matsuura, Kazuhiro Tanaka, Shuichi Matsuda, Yoshinao Oda, Yoshifumi Hori, Akira Yokomizo, Yukihide Iwamoto

**Affiliations:** 1 Department of Orthopedic Surgery, Graduate School of Medical Sciences, Kyushu University, Fukuoka, 812-8582, Japan; 2 Department of Anatomic Pathology, Graduate School of Medical Sciences, Kyushu University, Fukuoka, 812-8582, Japan; 3 Department of Urology, Graduate School of Medical Sciences, Kyushu University, Fukuoka, 812-8582, Japan

## Abstract

**Background:**

The skeletal muscle is an unusual site for metastasis from renal cell carcinoma (RCC). Metastatic RCC must be differentiated from benign primary soft-tissue tumors because aggressive surgical resection is necessary.

**Case presentation:**

We present the case of a 65-year-old man with metastatic RCC in the gluteus maximus muscle (3.8 cm in diameter) found on enhanced computed tomography (CT) 6 years after nephrectomy. Retrospectively, the small mass (1 cm in diameter) was overlooked 5 years earlier on enhanced CT. Because the growth of the lesion was slow, benign tumor was a differential diagnosis. However, magnetic resonance imaging (MRI) showed that the mass had high-signal intensity on T1- and T2-weighted images (WIs) compared to that of skeletal muscle, with mild enhancement by Gadolinium. The MRI features were unusual for most soft-tissue tumors having low-signal intensity on T1-WI and high-signal intensity on T2-WI. Therefore, under a diagnosis of metastatic RCC, the lesion was resected together with the surrounding skeletal muscle. The histology was confirmed to be metastatic RCC.

**Conclusion:**

MRI features of metastatic RCC may be beneficial in differentiating it from primary soft-tissue tumor.

## Background

Renal cell carcinoma (RCC) has widespread and unpredictable metastatic potential [1], even after curative nephrectomy is performed [2,3], RCC is able to metastasize to virtually any site. The most common sites of metastatic RCC are the lungs, lymph nodes, bones, liver and brain [4]. In several autopsy series, about 0.4% of cases with RCC had skeletal muscle metastases [2].

Making a diagnosis of metastatic RCC to the skeletal muscle is challenging, because the site is unpredictable, in addition to it being rare. Furthermore, cases of metastasis arising long after nephrectomy have been reported [2,5]. The differential diagnosis is primary soft-tissue tumor. It is particularly important that benign soft-tissue tumor should be differentiated, because aggressive surgical resection is necessary for metastasized RCC, but not for benign soft-tissue tumor [6].

Generally, either open or needle biopsy is necessary in order to make a diagnosis, in cases of soft-tissue tumor, because the surgical procedure is different depending upon the histological diagnosis. In the current paper, we present a case of RCC with metastasis to the gluteus muscle. The lesion was treated without biopsy, because the MRI features suggested metastatic RCC. We wish to emphasize the MRI features of metastatic RCC to the skeletal muscle, which could be beneficial in differentiating metastatic RCC from primary soft-tissue tumor.

## Case presentation

**Figure 1:**
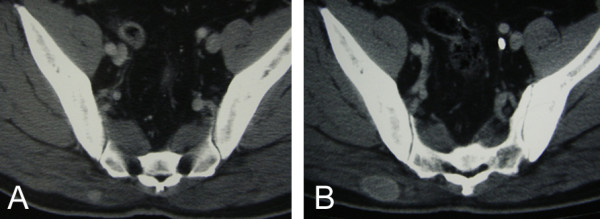
Computed tomography with contrast medium (100 ml, Omnipaque300, Daiichi-Sankyo, Japan) shows a well-defined mass in the gluteus maximus muscle, at the age of 60 years old (A; Toshiba, Aquilion^TM4^, 120 kV, 300 mA, 0.5 sec/r) and at the age of 65 years old (B; Toshiba, Aquilion^TM64^, 120 kV, 300 mA, 0.5 sec/r).

A 59-year-old man underwent right radical nephrectomy for RCC of 3.8 centimeters in diameter (stage pT1a N0 M0). One year after the nephrectomy (when he was 60 years old), recurrence was seen in the adrenal gland. Consequently, resection of the adrenal gland was undertaken. Six years after the nephrectomy (when he was 65 years old), a routine checkup using computed tomography (CT) of the chest and abdomen detected a small mass in the gluteus maximus muscle. The tumor was located in the gluteus maximus muscle adjacent to the fascia, and this was enhanced by contrast medium (Figure 1). On physical examination, the tumor was palpable in the gluteus maximus muscle. He had no tenderness or referred pain. Retrospectively, the tumor had been visible 5 years earlier, 1 year after the nephrectomy, as a mass of 1 cm in diameter (Figure 1). CT examination had not included the area of the gluteus maximus muscle until the latest CT examination.

**Figure 2:**
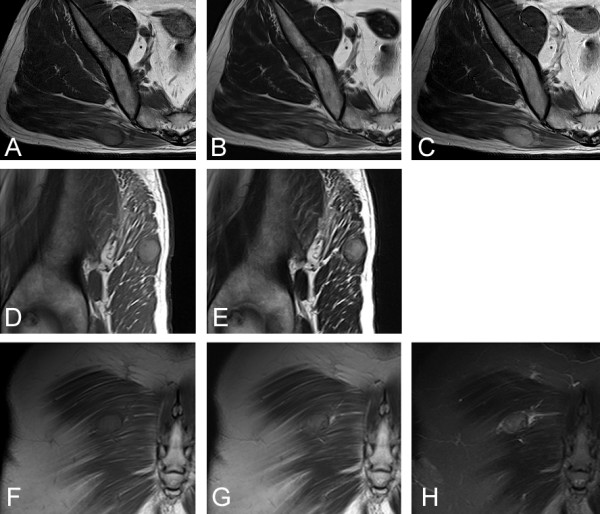
MRI shows diffuse high-signal intensity on T1- (A, D, F; TR/TE = 487/9.5) and T2- (B, E, G; TR/TE = 3500/89) weighted images with capsulation of low-signal intensity on T1- and T2-weighted images (A, B; axial, D, E; sagittal, F, G; coronal). Edema of low-signal intensity on T1-weighted and high-signal intensity on T2-weighted images can be observed (A, B). The tumor has high-signal intensity on T2-STIR (H; coronal). Moderate enhancement by Gadolinium (0.2 ml/kg, Magnebist, Schering, Germany) is seen on T1-weighted image (C; axial). (Siemens Symphony 1.5T, FOV = 200 × 200 mm, matrix = 256 × 320).

Magnetic resonance image (MRI) showed that the lesion adjacent to the fascia had higher signal intensity than that of skeletal muscle on T1- and T2-weighted, and T2-STIR images. The lesion had a rather regular border to the surrounding gluteus maximus muscle, and was capsulated by a thin area with low-signal intensity on both T1- and T2-weighted images (Figure 2A–2H). Edema of low-signal intensity on T1-weighted and high-signal intensity on T2-weighted images was observed (Figure 2A–2B). The lesion was mildly homogeneously enhanced by Gadolinium on T1-weighted imaging (Figure 2C).

**Figure 3:**
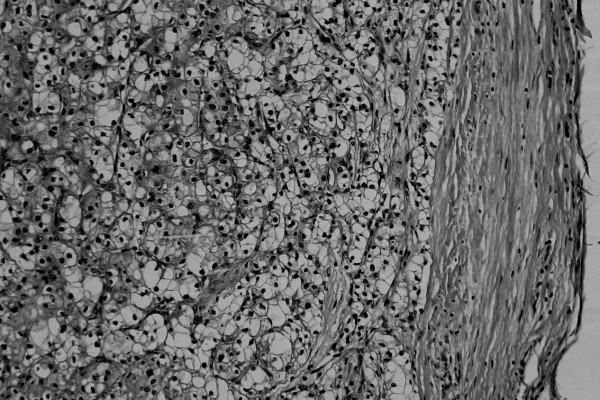
Typical morphology of a clear-cell renal cell carcinoma (Fuhrman grade 2), with small round-shaped nucleoli and abundant clear cytoplasm, with nests of tumor cells separated by thin-walled, sinusoidal vascular spaces (Hematoxylin and Eosin original magnification ×180).

Primary soft-tissue tumor was a differential diagnosis. Because the growth of the lesion was slow over 5 years, benign soft-tissue tumor was suspected. The fact that the lesion was capsulated by a thin area as seen on MRI also supported the diagnosis. However, the MRI features were unusual for most soft-tissue tumors having low-signal intensity on T1-weighted imaging and high-signal intensity on T2-weighted imaging. Therefore a diagnosis of metastatic RCC was suspected rather than primary soft-tissue tumor. Because metastatic RCC was strongly suspected, wide resection including the surrounding skeletal muscle was undertaken. Biopsy was not performed in order to avoid unnecessary contamination from the procedure. The resected tumor was composed of alveolar proliferation of carcinoma cells with clear cytoplasm and small round-shaped nucleoli (Fuhrman grade 2), resembling the previously resected RCC, suggesting metastatic RCC (Figure 3).

## Discussion

Surgical resection of metastatic RCC reportedly improves the outcome of patients with metastatic RCC, and five-year survival rates are between 35% and 50% after surgical therapy for solitary metastasis [3,7]. In the current case, metastatic RCC to the skeletal muscle was found by chance during a regular checkup for metastasis using CT on the abdominal cavity. The metastasis had been overlooked for several years. The lesion may have been detected earlier by scanning with a wider range, namely because there was a metastasis in the adrenal gland, and it may thus be preferable to always cover the area from the hepatic dome down to below the symphysis during CT follow-up. However, generally, it is difficult to detect metastases to the skeletal muscle, because the area of skeletal muscle to be metastasized can vary. Moreover, the tumors may be painless, and they may go unnoticed when they are small [8]. As for metastatic RCC to the gluteus maximus muscle, there have been several previous reports [9,10].

Another factor which makes a correct diagnosis difficult is that metastasis sometimes occurs long after primary surgical treatment. It has been reported that 11% of metastatic RCC cases occurred more than 10 years after initial diagnosis [1]. Distant metastasis to the parotid gland has been reported 17 years after primary surgery [5] and metastasis to the skeletal muscle has been reported 15 years after primary surgery [2]. Therefore, in order to reduce the chance of overlooking a malignant tumor, the possibility of metastasis should always be considered for patients with a history of RCC, even long after primary surgery.

Metastatic RCC to the skeletal muscle must be differentiated from primary soft-tissue tumors. Primary soft-tissue tumors are more common than metastatic tumors to the skeletal muscle [8]. Generally, either open or needle biopsy is necessary in order to make a diagnosis in cases of soft-tissue tumor. In cases where malignancy is proven by biopsy, the puncture tract should be excised in order to avoid tumor seeding. Dissemination due to needle biopsy is rare. However, this may not be true for possible hypervascular tumors as in the current case. Furthermore, needle biopsy has the risk that the tip of the needle may penetrate the tumor, when the tumor is small. MRI features of metastatic RCC to the skeletal muscle may show high-signal intensity on T1- and T2-weighted MRI [7], as seen in the current case. These signal intensities seem to be characteristic for metastatic RCC to the skeletal muscle, even in other previous reports where such points were not emphasized by the authors [8,11]. We did not perform a biopsy in the current case, since the MRI features of the tumor were compatible with metastatic RCC to the skeletal muscle.

In the current case, the characteristic features of MRI for metastatic RCC were beneficial in differentiating it from primary soft-tissue tumor, because most cases of primary soft-tissue tumor have low- to iso-signal intensity relative to the skeletal muscle on T1-weighted images and high-signal intensity on T2-weighted images. Although lipoma, hemangioma [12], clear cell sarcoma (malignant melanoma of the soft parts) [13] and alveolar soft-part sarcoma [14] are all known to have high-signal intensity on T1-weighted images, fat-suppression MRI is useful to differentiate lipoma. It has been reported that hemangioma can be differentiated from other malignant soft-tissue tumors by the existence of lobulation, septation, and central low-signal intensity [12]. On the other hand, clear cell sarcoma (malignant melanoma of the soft parts) and alveolar soft-part sarcoma may be difficult to differentiate from metastatic RCC on MRI. However, MRI may still be beneficial in excluding benign tumors of lipoma and hemangioma from malignant tumors of metastasis RCC, clear cell sarcoma or alveolar soft-part sarcoma.

The high intensity of hemangioma seems to be related with the fat component, as is the case with lipoma. Higher signal intensity on T1-weighted images of clear cell sarcoma (malignant melanoma of the soft parts) is reported to be correlated with melanocytic differentiation [13]. We have no reasonable explanation for the high intensity on T1-weighted image of alveolar soft-part sarcoma and RCC. The histological features of these two lesions have similar aspects of rich cytoplasm. Therefore, there may be some underlying common molecule, contributing to the high-signal intensity of T1-weighted images, although we have no way of knowing this as yet.

It has been reported that angiography shows that RCC metastatic to the skeletal muscle usually appears as hypervascular lesions [7,8]. The Gadolinium enhancement of MRI in the current case was mild. Gadolinium is a paramagnetic metal having a very efficient T1 relaxation mechanism. In order to be effective, Gadolinium needs to reach the tissue. Although the degree of enhancement depends upon the type of sequence and the delay after contrast injection, early dense venous drainage [8], due to an arteriovenous shunt in RCC [7] might reduce the time for Gadolinium to reach the tissue in order for it to be effective, resulting in mild enhancement. It is also possible that mild enhancement shows actually hypovascularity in the tumor, or perhaps gadolinium enhancement is simply not correlated with the degree of vascularity.

## Conclusion

In conclusion, we present the case of a 65-year-old man with metastatic RCC to the gluteus maximus muscle. The nodule was slowly progressive over 5 years. MRI of metastatic RCC is characterized by high-signal intensity on T1- and T2-weighted images which may be beneficial in the differential diagnosis from primary soft-tissue tumors. Maintaining a high degree of suspicion of metastatic RCC is required for patients with a history of RCC.

## Competing interests

The author(s) declare that they have no competing interests.

## Authors' contributions

AS drafted the manuscript. TY, KT and ShM participated in the design of the study and performed. SuM, YO and YH and conceived of the study. AY and YI participated in its design and coordination and helped to draft the manuscript. All authors read and approved the final manuscript. All authors read and approved the final manuscript.
